# Monthly Summary

**Published:** 1869-04

**Authors:** 


### MONTHLY SUMMARY.

*Employment of Glycerine of Tannin.*—Dr. Sydney Ringer, in an interesting paper (*The Practitioner*, July, 1856.) says that this preparation of tannin appears to be but little known, while in his opinion it is very serviceable in many diseases. In this country it is perhaps even less known. We do not find it mentioned in the U. S. Dispensatory; but on reference to a very recent English work (*Dictionary of Mat. Med. and Ther.*, by Adolph Wahltuch, M. D.) we find the following formula for its preparation: R.—Gallic acid, ʒ j; Glycerine, f ʒ iv. Rub and heat. Dr. R. thinks this preparation of great use in ozæna; in the thin sanious or thicker purulent discharge from the nostrils which sometimes occurs after measles, scarlatina, and other diseases; in the obstruction of the nose frequent in syphilitic children. The thin, sanious or purulent discharge from the ears so commonly met with in unhealthy children, can, he says, be stopped at once by filling up the external meatus with this preparation. If there be acute inflammation of the meatus, this thould be relieved before using the tannin.

In many cases of *eczema* he says this preparation is of very great use. " It is of service only in the earlier stages of the disease. Thus, when the skin is inflamed, red, swollen, and weeping, if the scabs be thoroughly removed, and the raw surface be painted over with this preparation of tannin, the discharge is stayed, the redness, heat, and swelling much lessened or removed, and the appearance of the parts much improved. When in a less active condition, and when the tissues are less red, swollen, and weeping, the eczema may more profitably be treated in the same way. The tissues assume a much healthier appearance, and after a few applications look like a healthy, healing sore. A poultice may be usefully applied at night, and this glycerine of tannin twice or three times in the day. All the advantages which accrue from its employment in this disease have not yet been mentioned, for the troublesome itching, and tingling, and burning, so common in eczema, are at once removed by this application, and thus the tearing with the nails and rubbing with the hands which prevents the healing of the sore, and causes it even to spread, is prevented, and the comfort and well doing of the patient much promoted, as the itching and feeling of burning often greatly breaks the sleep. Sometimes the glycerine of tannin does not, of itself, quite remove the disease, but brings it to the stage where there is only a little desquamation, with a tendency to crack and ooze. It may be necessary in such case to perfect the cure by a resort to tar or carbolic acid ointment. It need not be said that some cases prove incurable by this as by all other treatment. Impetigo may be beneficially treated in the same way. The scabs should be removed by a poultice applied each night, while this tannin preparation is employed during the day. In the treatment of these diseases of the skin by this application, the state of the digestive organs must not be overlooked, but anything wrong with them should, if possible, be removed.

"The eczema which occurs behind the ears of children, and is often limited to these places, is most admirably treated with the remedy. It almost always dries up and heals after one or two applications, even when it has lasted for weeks or for months. The gums, if red and swollen, should be lanced, or other irritations removed. Intertrigo of children may also be treated in this way."

He thinks it an extremely useful application to the throat in chronic inflammation and in superficial ulceration of the pharyngeal mucous membrane, and when this membrane is relaxed, moist, and granular looking, etc.—*Am. Jour. Med. Sciences*

---

*Mode of Dividing Glass.*—The following plan, to break a bottle or jar across its circumference, so as to form a battery cup or vessel for other purposes, may be of some service to your readers. I have performed the operation successfully many times. Place the bottle in a vessel of water, to the height where it is designed to break it; also fill the bottle to the same level. Now pour coal oil inside and out on the water; cut a ring of paper, fitting the bottle. Saturate with alcohol or benzine, so that it touches the oil. Pour, also, some inside the bottle. Set on fire; the cold water prevents the glass from heating below its surface, while the expansion caused by the heat will break the vessel on the water line.—J. T. Peet, *Scientific American.*

*Steel.*—Steel is successfully alloyed with other metals, improving its qualities for some purposes. One five hundredth part of silver adds immensely to the hardness of the steel and yet increases its tenacity. One hundredth part of platinum, though not forming so hard an alloy as the silver and steel gives a very great degree of toughness. Rhodrum, pallidum, irridum, and osium, make steel very hard, but their use, from their cost, is confined mainly to the experimental laboratory.—*The Druggist's Circular and Chemical Gazette.*

*Fracture by Ointment.*—Mr. B. W. Switzer, Asst. Surg. 6th Punjaub Infantry, relates (*Med. Times and Gaz.*, Sept. 19, 1868) the following curious case. A Hindoo boy, aged about four years, was brought to him for treatment. On examination, the right humerus was found to present a uniform enlargement, from about three inches below the head to within two inches of the condyles, tapering above and below. The history of the case was that the boy had been running from something that frightened him, and fell heavily on his arm, sustaining a bad comminuted simple fracture through the whole extent of the middle third of the bone. Residing far from any surgical aid, his people simply let him alone, and this tumor was but nature's rough surgery. No trace of crepitus remained ; it was from head to condyle one solid bone. Such was his state eight months after the accident. Mr. S. was at a loss what to do ; he allowed the boy to run about, and his general health improved, and finally, for the sake of doing something, he ordered him an ointment containing 100 grains of iodide of potassium and 10 grains of iodine to an ounce of lard, to be rubbed into the tumor twice a day ; he was also to take internally a grain of the iodide twice daily. After treatment of this kind for about three weeks, on examining the arm crepitus was detected and the tumor found, like an iceberg in summer, rapidly breaking up in every direction, Finally all the callus was absorbed and the fragments left movable. All medicine was then stopped, and the bone properly set in splints. He made a capital recovery, callus being again thrown out ; and the fragments re-united in their proper places. *Med. News and Library,*

*Early Dentition.*—M. Guenoit related to the Societe de Chiurgie the case of an infant which, when nine days old, exhibited a spontaneuos expulsion of the two middle upper incisor teeth, together with the destruction and expulsion of the dental bulb. There was some gingival stomatitis, but no abscess of any kind. The teeth resembled two solid shells, covered with a thin layer of enamel. These cases are rare. In connection with this subject M. Guenoit enumerated several celebrated persons who who are said to have been born with teeth, such as Mirabeau, Mazarin, Louis XIV., to which he would have added that of M. Broca had not this gentleman disclaimed any right to such a distinction. Believing the fact generally admitted, that infants are occasionally born with teeth ready cut, we are greatly surprised to find such an experienced accoucheur as M. Blot utterly denying its accuracy. He says he has never met with an instance of its occurrence in 30,000 infants that have come under his observation, and the experience of his colleagues is just as negative. However, that unfailing repertory of information, M. Giraldes, was enabled to refer to numbers of cases of children born with one or more teeth; and he has met with similar cases in his own practice. M. Besneir observes that such cases are familiar enough to matrons, who are in the habit of at once extracting the teeth. We suspect that this operation must have been already performed in cases that otherwise would have attracted M. Blot's attention.—*Med. Times and Gazette,*

---

*Extraordinay Cure Of Epilepsy.*—A most extraordinary case was communicated to the Surgical Society of Ireland by Dr. Kirhead, of Tuam, at their last meeting, the details of which we shall lay before our readers in the reports of the Society next week. A patient had been subject to epileptic fits and had been treated without benefit for them. Being taken suddenly in one of the attacks, the patient fell with the head against the bars of the grate, and sustained very severe burns over the parietal bones. After a protracted illness, the parietal bone became detached and exfoliated almost entire, and the patient recovered, cured of the epilepsy but minus the parietal bone, and with no protection for the brain but the cicatrized integument.—*Dublin Med. Press & Cir.*

*The Stains of Iodine.*—Add a few drops of liquid carbolic acid to the iodine tincture, and the latter will not stain. Dr. Bogs, of the Indian Service, states that the carbolic acid, besides the above mentioned property, renders the efficacy of tincture of iodine more certain. Whenever injections of the latter are indicated, he advises the following formula: Alcoholic tincture of iodine, one ounce (this proportion is mentioned by the *France Medicale*, but there is evidently a mistake—instead of "30 grammes" it should probably be "3 grammes,"—viz., 45 drops); pure liquid carbolic acid, six drops; glycerine, one ounce; distilled water, five ounces, This is said to be far superior to tar-water in blennorrhœa and leucorrhœa.—*Lancet*, Dec. 12, 1868.

---

*Second Sight.*—There recently died in Pensacola, Florida, an old colored Preacher, known as "Uncle Jacob," about one hundred years old. He was present at and could distinctly remember the surrender of *Cornwallis* at Yorktown. A curious fact occurred in his case, the truth of which we have vouched for by a respectable medical gentleman there. It is, that after many years of total blindness, this old colored man, over one hundred years of age, was astonished to awake one morning and find his eyesight fully restored—this occurred recently, and the old mau retained his eyesight up to the hour of his death. *Med. & Surg. Reporter.*

---

